# Hearing Phenotypes of Patients with Hearing Loss Homozygous for the *GJB2* c.235delc Mutation

**DOI:** 10.1155/2020/8841522

**Published:** 2020-08-01

**Authors:** Chang Guo, Sha-Sha Huang, Yong-Yi Yuan, Ying Zhou, Ning Wang, Dong-Yang Kang, Su-Yan Yang, Xin Zhang, Xue Gao, Pu Dai

**Affiliations:** ^1^College of Otolaryngology Head and Neck Surgery, Chinese PLA General Hospital, Chinese PLA Medical School, 28 Fuxing Road, Beijing 100853, China; ^2^National Clinical Research Center for Otolaryngologic Diseases, State Key Lab of Hearing Science, Ministry of Education, China; ^3^Beijing Key Lab of Hearing Impairment Prevention and Treatment, Beijing, China; ^4^Department of Pediatric Surgery, Chinese PLA General Hospital, Chinese PLA Medical School, 28 Fuxing Road, Beijing 100853, China; ^5^Department of Otolaryngology, PLA Rocket Force Characteristic Medical Center, 16# XinWai Da Jie, Beijing 100088, China

## Abstract

Hereditary hearing loss is characterized by remarkable phenotypic heterogeneity. Patients with the same pathogenic mutations may exhibit various hearing loss phenotypes. In the Chinese population, the c.235delC mutation is the most common pathogenic mutation of *GJB2* and is closely related to hereditary recessive hearing loss. Here, we investigated the hearing phenotypes of patients with hearing loss associated with the homozygous c.235delC mutation, paying special attention to asymmetric interaural hearing loss. A total of 244 patients with the *GJB2* c.235delC homozygous mutation encountered from 2007 to 2015 were enrolled. The severity of hearing loss was scaled with the American Speech-Language-Hearing Association (ASHA). Auditory phenotypes were analyzed, and three types of interaural asymmetry were defined based on audiograms: Type A (asymmetry of hearing loss severity), Type B (asymmetry of audiogram shape), and Type C (Type A plus Type B). Of the 488 ears (244 cases) examined, 71.93% (351) presented with profound hearing loss, 14.34% (70) with severe hearing loss, and 9.43% (46) with moderate to severe hearing loss. The most common audiogram shapes were descending (31.15%) and flat (24.18%). A total of 156 (63.93%) of the 244 patients exhibited asymmetric interaural hearing loss in terms of severity and/or audiogram shape. Type A was evident in 14 of these cases, Type B in 106, and Type C in 36. In addition, 211 of 312 ears (67.63%) in the interaural hearing asymmetry group showed profound hearing loss, and 59 (18.91%) exhibited severe hearing loss, with the most common audiogram shapes being flat (27.88%) and descending (22.12%). By contrast, in the interaural hearing symmetry group, profound hearing loss was observed in 140 ears (79.55%), and the most common audiograms were descending (46.59%) and residual (21.59%). Hearing loss associated with the *GJB2* c.235delC homozygous mutation shows diverse phenotypes, and a considerable proportion of patients show bilateral hearing loss asymmetry.

## 1. Introduction

Hearing loss (HL) is one of the most common neurosensory impairments in humans [1]. The World Health Organization estimated in 2020 that over 5% of the world's population (approximately 466 million people) suffer from HL [2]. Sensorineural HL (SNHL) is the most common form of HL and typically is caused by a loss of functional sensory hair cells (HCs) and supporting cells (SCs) within the cochlea [3, 4]. HCs and SCs develop from common progenitor cells within the prosensory domain of the developing cochlea [5]. HCs transfer mechanical vibration into an acoustic electrical signal, which is then transmitted to the auditory cortex via spiral ganglion neurons (SGNs). SCs are important cells that support HCs and hold the potential to regenerate HCs once damaged [6–8]. HCs and SCs are very sensitive and vulnerable to stress and damage, classified mainly as genetic factors, environmental factors, ototoxic drugs, aging, inflammation, and other unknown etiologies [9–11]. Among these, genetic factors are responsible for approximately 50-60% of cases of HL [12]. Whereas 70% of genetic HL cases are nonsyndromic HL (NSHL), 30% are syndromic. Approximately 80% of NSHL cases are inherited via an autosomal recessive mode, whereas other cases are inherited in an autosomal dominant, X-linked, or mitochondrial mode [13, 14]. To date, more than 100 genes have been shown to cause NSHL [15]. Despite this, the most common cause of NSHL is mutations in *GJB2* [16, 17].

The *GJB2* gene encodes a 26 kDa gap junction protein known as connexin 26 (Cx26) [18]. Cx26 consists of an N-terminal helix, four transmembrane helices (TM1-4), two extracellular loops (E1 and E2), a cytoplasmic loop (CL), and a C-terminus [19]. Cx26 is expressed in the inner ear, which contains SCs, stria vascularis, spiral ligament, and spiral limbus [20]. Cx26 is associated with proteins that form a transmembrane hexameric gap junction channel known as a connexon. These channels are believed to play a role in the recycling of potassium ions from HCs to the endolymph [21, 22]. *GJB2* is involved in a series of physiological hearing processes including cochlear development, endocochlear potential generation, active cochlear amplification, and second messenger transduction [23, 24].

The *GJB2* mutation is one of the most common pathogenic factors related to genetic HL, and *GJB2* usually is the first deafness gene to be evaluated during clinical diagnosis due to the observations that *GJB2* is the most common human deafness gene in almost all populations studied so far. Currently, more than 300 mutations in *GJB2* have been reported (the Human Gene Mutation Database) [25]. Notably, several alleles have been found to be particularly enriched in certain populations: c.35delG in Europe, America, North Africa, and the Middle East; c.71G>A in India and Pakistan; c.167delT in Ashkenazi Jews; and c.109G>A in East and Southeast Asia [16, 26–28]. The contribution of *GJB2* mutations to genetic HL varies by ethnicity, but such mutations are the primary cause of congenital severe-to-profound autosomal recessive NSHL (up to 50% worldwide) [29, 30]. In addition, mild and moderate HL are associated with *GJB2* common mutations such as c.35delG and c.109G>A, showing diverse hearing phenotypes [31, 32]. In the Chinese population, the most common *GJB2* mutation is c.235delC (68.9%) [33]. Base deletion creates a frameshift mutation, and early termination of translation yields a nonfunctional protein [34]. Although severe-to-profound HL is the most common clinical presentation of patients with *GJB2* c.235delC, various hearing phenotypes have been reported, and the HL caused by the mutation exhibits clinical heterogeneity [35, 36].

Understanding genotype-phenotype relationships will provide novel insights into molecular diagnosis, genetic counseling, and genetic therapy. Many approaches, such as cochlear implant surgery, gene therapy, and cell therapy, have been used to treat *GJB2*-related HL [37–39]. Since 2009, preimplantation genetic diagnosis (PGD), an effective method to prevent the recurrence of genetic HL, has been successfully applied to protect babies against *GJB2*-related HL [40, 41]. We believe that more treatment options will be available for these patients in the future. Here, we analyzed audiological data of 244 patients with *GJB2* c.235delC homozygous mutation-induced HL and explored the phenotypic diversity of HL in patients with *GJB2* c.235delC with a focus on the symmetry (or lack thereof) of binaural hearing.

## 2. Materials and Methods

### 2.1. Clinical Data

Patients for whom complete audiological data were available and who visited the molecular diagnostic center of the Chinese PLA General Hospital from 2007 to 2015 were included. The inclusion criteria were (1) binaural SNHL with a complete hearing history, data from physical examination and a detailed ENT examination, and audiological test results; (2) DNA sequence of patients were confirmed to have *GJB2* c.235delC homozygous mutation; and (3) no syndromic HL or ear-related diseases (e.g., acute or chronic otitis media, advanced Meniere's disease, acoustic neuroma, meningoencephalitis, or trauma). A total of 244 subjects (130 males and 114 females) were included.

### 2.2. Research Methods

#### 2.2.1. Genomic DNA Extraction

Peripheral blood samples were collected from arm veins, and the genomic DNA of leukocytes was extracted [42].

#### 2.2.2. Detection of Mutations in the Coding Region of *GJB2*

We performed primer design, PCR amplification, and direct sequencing using the methods descrbied by Dai et al. [43]. We compared the results to the wild-type sequence (*GJB2*: NM_004004) using GeneTool Lite ver. 1.0.

#### 2.2.3. Audiologic Evaluation

Audiological tests were performed in the hearing center of the Chinese PLA General Hospital. Tests included pure-tone audiometry (or behavioral audiometry) for patients > 4 years old and multiple-frequency auditory steady-state evoked response (ASSR) tests for patients ≤ 4 years old.

*(1) HL Severity*. We derived the average air conduction (AC) pure-tone hearing thresholds or ASSR response thresholds at 0.5, 1, 2, and 4 kHz for both ears. If data could not be obtained at any frequency using the maximal output, that output was taken to be the hearing or response threshold. Using the American Speech-Language-Hearing Association (ASHA) [44], we scaled hearing as normal (threshold: ≤25 dB), mild HL (threshold: 25.1-40 dB), moderate HL (threshold: 40.1-55 dB), moderately severe HL (threshold: 55.1-70 dB), severe HL (threshold: 70.1-90 dB), and profound HL (threshold: >90 dB, including total deafness). According to WHO criteria [45], disabling HL referred to HL greater than 40 dB in the better hearing ear in adults and HL greater than 30 dB in the better hearing ear in children (age ≤ 15 years old). HL progression was defined as an elevation of the average hearing or response thresholds by >15 dB in one or both ears between audiograms. Only patients with multiple audiograms and at least a 4-month gap between audiograms were included in the analysis of progressive HL [46].

*(2) Audiogram Shapes*. We recognized seven shapes of pure-tone threshold audiograms: descending (≥15 dB HL difference between the [better] average thresholds at 250 and 500 Hz and those at 4,000 and 8,000 Hz), flat (≤15 dB HL difference between all thresholds from 125 to 8,000 Hz), valley like (≥10 dB HL difference between the poorest midfrequency [1,000-2,000 Hz] threshold and those at higher and lower frequencies), ascending (≥15 dB HL difference between the average thresholds at 250 and 500 Hz and the [better] average thresholds at 4,000 and 8,000 Hz), residual (residual hearing at only one or two frequencies), total deafness (no hearing at any frequency when outputs are maximal), and unclassified (none of the above). We defined descending, flat, valley like, ascending, residual, and total deafness shapes as “regular” audiograms.

We recognized seven shapes of ASSR audiograms: descending (≥15 dB HL difference between the [better] mean thresholds at 500 and 1,000 Hz and those at 2,000 and 4,000 Hz), flat (≤15 dB HL difference between all thresholds from 500 to 4,000 Hz), valley like (≥10 dB HL difference between the poorest midfrequency [1,000–2,000 Hz] threshold and those at higher and lower frequencies), ascending (≥15 dB HL difference between the mean thresholds at 500 and 1,000 Hz and the [better] mean thresholds at 2,000 and 4,000 Hz), residual (residual hearing at only one or two frequencies), total deafness (no hearing at any frequency when outputs are maximal), and unclassified (none of the above). Similarly, we defined the first six shapes of audiograms as “regular” audiograms.

**Figure 1:**
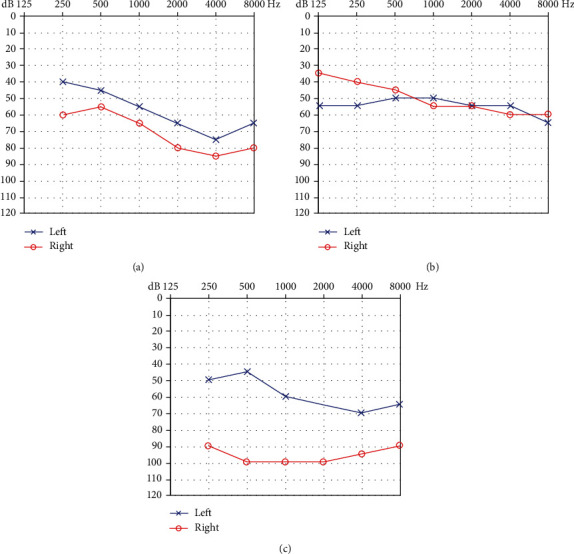
(a) Audiogram of asymmetry of HL severity (Type A asymmetry). (b) Audiogram of asymmetry of audiogram shape (Type B asymmetry). (c) Audiogram of asymmetry of hearing loss severity and audiogram shape (Type C asymmetry). In all audiograms, the frequency in hertz (Hz) is plotted on the *x*-axis and the hearing level in decibels (dB HL) on the *y*-axis.

*(3) Asymmetric Hearing Phenotypes*. *(1) Asymmetry of HL severity (Type A asymmetry)*: binaural audiograms were shaped similarly, but hearing thresholds differed, with an HL difference ≥ 10 dB at a minimum of four frequencies, an HL difference ≥ 15 dB at two frequencies, or an HL difference ≥ 25 dB at one frequency (Figure 1(a)).

*(2) Asymmetry of audiogram shape (Type B asymmetry)*: HL on either side was similar (average hearing threshold difference ≤ 15 dB) but audiogram shapes differed, and at least one ear exhibited a regular audiogram (e.g., a descending type in one ear but a flat type in the other; Figure 1(b)).

*(3) Asymmetry of HL severity and audiogram shape (Type C asymmetry)*: the average hearing threshold difference was >15 dB. In addition, if the audiogram shapes were different, at least one ear exhibited a regular audiogram. If the two audiograms were irregular, the binaural hearing threshold difference was considered asymmetric. Alternatively, if the hearing threshold difference was ≥15 dB at two frequencies from 0.125–8 kHz, or ≥10 dB at three frequencies, there was likely Type C asymmetry (Figure 1(c)).

## 3. Results

### 3.1. Patient Demographics

The patients ranged in age from 3 months to 45 years: 138 patients were 0–4 years, 60 were 5–71 years, and 46 were 18–45 years. Definite ages of onset were identified in 166 patients and ranged from birth to 24 years (average: 1.9 years). Note that six patients were siblings from three families (two per family); the remaining patients were sporadic cases.

### 3.2. HL Severity

The HL severity (both ears) for all 244 patients is shown in Table 1. Profound HL was most common (71.93%, 351/488), followed by severe HL (14.34%, 70/488) and moderately severe HL (9.43%, 46/488). Fewer patients exhibited moderate HL (4.10%, 20/488) or mild HL (0.2%, 1/488). All patients showed disabling HL. Only five patients met the criteria for inclusion in the progressive HL analysis, but none of them had progressive HL.

### 3.3. Audiograms

Among the 244 cases (488 ears), descending (30.94%, 151/488) and flat (24.39%, 119/488) were the most common audiogram shapes, followed by residual (15.57%, 76/488), total deafness (5.53%, 27/488), valley like (5.33%, 26/488), and ascending (2.05%, 10/488). The unclassified shape constituted 16.19% (79/488) of all audiograms.  Table 2 shows that 40.16% (98/244) of cases exhibited the same (regular) audiogram shape in both ears.

### 3.4. Interaural Hearing Asymmetry

A total of 88 of the 244 cases were symmetric in terms of both HL severity and audiogram shape. By contrast, 156 (63.93%) cases were asymmetric in terms of audiogram shape and/or HL severity (14 Type A, 106 Type B, and 36 Type C). In the latter group, 211 ears (67.63%) exhibited profound HL and 59 (18.91% of all ears) exhibited severe HL. In patients with symmetric HL, 140 ears (79.55%) exhibited profound HL (Table 3).

Of the 156 patients with asymmetric interaural HL, 87 ears (27.88%) exhibited flat, 69 (22.12%) descending, and 37 (11.86%) residual audiograms. Of the 88 cases with symmetric interaural HL, 82 ears (46.59%) exhibited descending, 38 (21.59%) residual, and 32 (18.18%) flat audiograms (Table 4).

### 3.5. HL Severity and Audiograms in Different Age Groups

We categorized age into four groups: group 1 (≤6 years old), group 2 (6.1–12 years old), group 3 (12.1–18 years old), and group 4 (≥18 years old). There were 156, 29, 17, and 42 patients in groups 1, 2, 3, and 4, respectively.

HL severity (both ears) in the different age groups is shown in Table 5. Among all groups, profound HL was the most common type of HL. In groups 1 and 4, severe HL was the second most common type of HL, followed by moderately severe and moderate HL. By contrast, in group 2, moderately severe and moderate HL were the second most common types of HL, followed by severe HL. Similarly, in group 3, moderately severe HL was the second most common type of HL, and a smaller proportion of patients showed moderate and severe HL. However, only one patient in group 1 exhibited mild HL.

The audiograms (both ears) in the different age groups are shown in Table 6. In group 1, the most common audiogram shape was flat (28.53%). In groups 2-4, descending was the most common audiogram shape (50%–61.76%). Among all groups, total deafness was relatively rare.

The interaural hearing asymmetry in the different age groups is shown in Table 7. Whereas Type B asymmetry (50.64%) was the most common type in group 1, symmetry was the most common in groups 2–4 (~41–53%). In groups 1–3, Type A asymmetry was the least common, whereas in group 4, Type C asymmetry was the least common.

### 3.6. HL in Three Sets of Siblings

**Figure 2:**
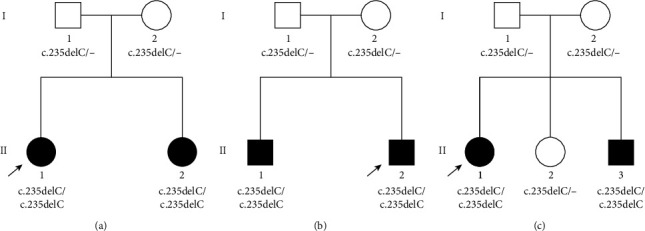
(a–c) are genotypes of the pedigrees for sets 1, 2, and 3, respectively.

**Figure 3:**
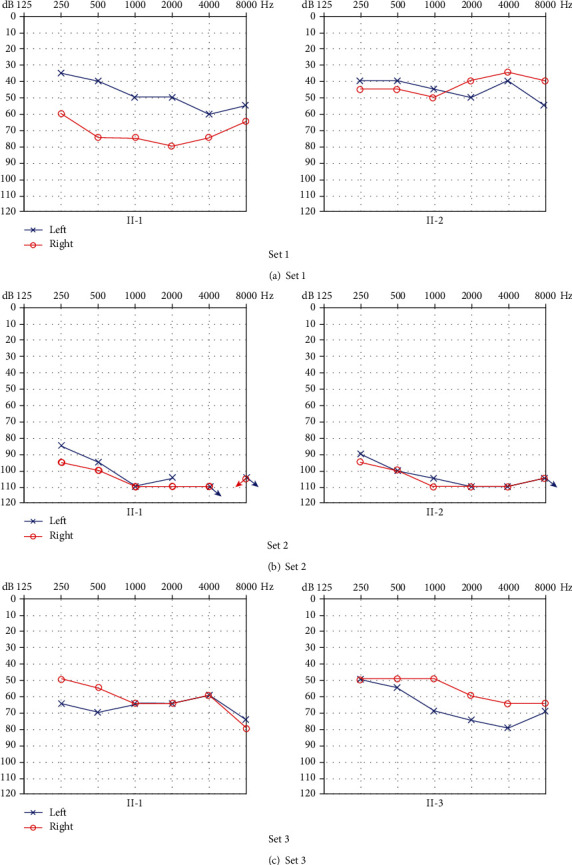
(a) Audiograms of two sisters (II-1 and II-2) in set 1. (b) Audiograms of two brothers (II-1 and II-2) in set 2. (c) Audiograms of the older sister (II-1) and younger brother (II-3) in set 3.

Set 1 was composed of two sisters (Figure 2(a)). Their normal-hearing parents were heterozygous carriers of c.235delC. The older sister (II-1) suffered from moderate and severe HL in the left and right ears. The younger sister (II-2) suffered from binaural moderate HL. The audiograms of the older sister were descending and unclassified (one ear each). The audiograms of the younger sister were flat in both ears. The older sister exhibited Type C asymmetric HL, and the younger sister exhibited symmetric HL. Set 2 was composed of two brothers (Figure 2(b)). Their normal-hearing parents were heterozygous carriers of c.235delC. Both brothers (II-1 and II-2) presented with binaural profound HL. The audiograms of the elder brother were descending in both ears. The audiograms of the younger brother were descending and flat (one ear each). The elder brother exhibited symmetric HL, and the younger brother exhibited Type B asymmetric HL. Set 3 was composed of an older sister and a younger brother (Figure 2(c)). Their parents and sister were all heterozygous carriers of c.235delC and had no hearing problems. Both siblings (II-1 and II-3) exhibited similar binaural moderate to severe HL with descending and flat audiograms (one of each). Both exhibited Type B asymmetric HL (Figure 3 and Table 8).

## 4. Discussion

HL has become a global public health problem. In addition to impaired communication, HL is associated with language delays, social adaptation problems, and even dementia [47]. Hereditary SNHL is genetically heterogeneous, and pathogenic mutations have been identified in approximately 50–60% of cases [48]. *GJB2* mutation is a major cause of hereditary NSHL, and most mutations are located in the coding region [49]. Up to 50% of cases of autosomal recessive NSHL are attributable to *GJB2* mutation in many populations worldwide [29, 30]. Therefore, genetic testing for *GJB2* mutations is a primary screening process for the molecular diagnosis of HL. As we mentioned previously, the *GJB2* mutation spectrum varies ethnically and geographically. In the Chinese population, the c.235delC homozygous mutation is the most common mutation in *GJB2* [33]. This gene encodes the connexin 26 (Cx26) protein. The gap junction proteins of adjacent cells allow for the exchange of information and materials; electrolytes, second messengers, and metabolites move through these channels, underlying both intercellular communication and homeostasis of the cochlear fluids, endolymph, and perilymph [50, 51]. Mutations in the *GJB2* coding region can cause frameshifts affecting protein translation and gap junction protein structure, resulting in a defective protein [52]. The *GJB2* c.235delC mutation causes early termination of translation and produces a nonfunctional Cx26 protein. The null Cx26 can induce apoptosis and oxidative damage in the cochlear duct, reduce the release of glutathione from connexin hemichannels, and decrease nutrient delivery to the sensory epithelium via cochlear gap junctions, thereby leading to HL. Cx26-deficient mouse models showed congenital HL and cochlear developmental disorders [53–55]. *GJB2*-related HL is usually binaural [42, 56], as in all 244 patients in this study. Profound HL was the most common (71.93%, 351/488) HL in our patients, followed by severe (14.34%, 70/488), moderately severe (9.43%, 46/488), and moderate (4.10%, 20/488) HL. Mild HL was seen in only 0.2% (1/488) of cases. Zhao et al. [35] retrospectively analyzed the hearing phenotypes of a large group of Chinese patients with HL caused by *GJB2* c.235delC biallelic mutations and found that most patients exhibited severe or profound HL, with only a few showing moderate HL. We also found that profound HL was most common; however, a considerable proportion of our cases exhibited moderate (4.10%) or moderately severe (9.43%) HL. HL phenotypes thus differ. All patients in our study showed disabling HL, indicating that *GJB2*-related HL usually has a negative impact on quality of life, ability to listen in noisy environments, communication with others, and comprehension ability, regardless of whether hearing in the two ears is symmetric. In the analysis of progressive HL, all five patients who met the criteria showed stabilization of HL. Our results were consistent with those of Chorath et al. [46], who concluded that progression of *GJB2*-related HL was rare. Unfortunately, the number of patients included was limited, and more follow-up data are required in future studies. Some researchers use the following definitions to classify audiograms [57, 58]: descending (>15 dB HL difference between the [better] average thresholds at 500 and 1,000 Hz and those at 4,000 and 8,000 Hz), flat (<15 dB HL difference between all thresholds from 250 to 8,000 Hz), midfrequency U-shaped (>15 dB HL difference between the poorest midfrequency [1,000–2,000 Hz] thresholds and those of higher and lower frequencies), and ascending (>15 dB HL difference between the average thresholds at 500 and 1,000 Hz and the [better] thresholds at 4,000 and 8,000 Hz). However, in our clinic, we found that the results yielded using these definitions sometimes do not agree with the characteristics of the audiograms. Thus, we amended the definitions. First, we changed the difference in dB HL from >15 dB to ≥15 dB for both the descending and ascending types, from <15 dB to ≤15 dB for the flat type, and from >15 dB to ≥10 dB for the valley-like type. Second, we used 250 and 500 Hz (not 500 and 1,000 Hz) as the low frequencies for the descending and ascending types and 2,000 and 4,000 Hz (not 4,000 and 8,000 Hz) as the high frequencies when evaluating ASSR data. In addition, when defining the flat type of ASSR, we use frequencies from 500 to 4,000 Hz. Third, we consider the residual and total deafness types to be “regular” types. We found that the descending (30.94%) and flat (24.39%) types were the most common, followed by the residual (15.57%), total deafness (5.53%), valley-like (5.33%), and ascending (2.05%) types. Our findings are similar to those of King et al. [57] in that most audiograms were descending, flat, or residual.

The phenotypes of genetic HL remain poorly understood. We analyzed the audiograms of 244 cases with homozygous *GJB2* c.235delC-associated HL. No consensus definition of “asymmetry” in the context of binaural HL has emerged. Early studies [59–61] proposed that an interaural difference(s) in pure-tone thresholds ≥ 10 dB at two frequencies or ≥15 dB at one frequency constitutes asymmetric HL. In a 2007 audiogram classification system (AMCLASS) [62, 63], audiograms were considered asymmetric if at least three frequencies differed by ≥10 dB, two by ≥15 dB, or one by ≥20 dB over the range of 0.25-8 kHz. In 2009, Mazzoli et al. [64] defined asymmetric HL as differences > 10 dB for at least two frequencies. These criteria concern only the severity of HL, not the audiogram shape; the picture is thus incomplete. To better characterize asymmetry, we divided it into three types with reference to both audiogram shape and HL severity. A substantial proportion of our cases with binaural HL exhibited interaural asymmetry in terms of audiogram shape and/or HL severity. A total of 156 (63.93%) patients exhibited asymmetric HL: 14 in terms of HL severity (Type A), 106 in terms of audiogram shape (Type B), and 36 with both Type A and B features (Type C). Our figures differ significantly from those of Wang et al. [36], who found a rate of asymmetric HL of 37.37% in children with biallelic protein-truncating mutations. This difference is probably attributable to variation in the definitions of binaural asymmetric HL. It is clear, however, that the *GJB2* c.235delC mutation is associated with significant variation in the binaural HL phenotype, evidencing a high level of genetic heterogeneity.

Among our 156 patients exhibiting interaural HL asymmetry, 211 ears (67.63%) suffered profound HL and 59 (18.91%) suffered severe HL. By contrast, in the 88 cases evidencing symmetric interaural HL, 140 ears (79.55%) showed profound HL. Thus, HL in such patients is likely to be profound or severe regardless of symmetry or asymmetry. It is worth noting that in patients with symmetric interaural HL, daily communication with others will be challenging since most suffered from bilateral profound HL. Among patients with asymmetric HL, audiograms were (in order) flat (27.88%), descending (22.12%), and residual (11.86%). By contrast, among patients with symmetric HL, audiograms were (in order) descending (46.59%), residual (21.59%), and flat (18.18%). Such subtle differences may be attributable to variation in HL severity. In all groups of different ages, profound HL was most common. HL severity tended to be more serious in group 1 (≤6 years old) and group 4 (≥18 years old). Among the patients < 18 years old, the most common audiogram shapes were descending and flat, whereas among the adult patients, the most common audiogram shapes were descending and residual. The differences may also be attributable to variation in HL severity. Among patients ≤ 6 years old, Type B asymmetry was the most common. However, among patients > 6 years old, symmetry was the most common. In three sibling pairs, HL severity was similar but audiogram shapes differed. Asymmetry may be attributable to one or more of heredity, epigenetics, and/or the environment. However, the mechanism of *GJB2*-related HL remains unclear. More clinical data, combined with full-exome and whole-genome sequencing, are needed.

## 5. Conclusion

A considerable proportion of patients homozygous for the *GJB2* c.235delC mutation exhibit significant variation in their binaural HL phenotypes, reflecting a high degree of bilateral HL asymmetry.

## Figures and Tables

**Table 1:** HL severity (both ears).

HL severity		Worse ear					Cases
		Mild	Moderate	Moderate to severe	Severe	Profound	
Better ear	Mild	*0*	0	0	1	0	1
	Moderate		*5*	7	3	0	15
	Moderate to severe			*11*	10	7	28
	Severe				*15*	26	41
	Profound					*159*	159

Italics indicate patients with the same HL severity in both ears.

**Table 2:** Audiogram shapes of both ears.

Audiogram shape		The other ear						
		Descending	Flat	Residual	Valley like	Ascending	Total deafness	Other
One ear	Descending	*50*	21	8	6	1	2	13
	Flat		*21*	12	8	4	3	29
	Residual			*19*	1	0	5	12
	Valley like				*1*	1	1	7
	Ascending					*1*	0	2
	Total deafness						*6*	4
	Other							*6*

Italics indicate patients with the same audiogram shape in both ears.

**Table 3:** HL severity in patients with asymmetric and symmetric interaural HL.

HL severity		Worse ear					Cases
		Mild	Moderate	Moderate to severe	Severe	Profound	
Better ear	Mild	*0 (0)*	0 (0)	0 (0)	0 (1)	0 (0)	0 (1)
	Moderate		*4 (1)*	4 (3)	0 (3)	0 (0)	8 (7)
	Moderate to severe			*4 (7)*	1 (9)	0 (7)	5 (23)
	Severe				*4 (11)*	2 (24)	6 (35)
	Profound					*69 (90)*	69 (90)

(1) The value outside/inside each pair of parentheses represents the number of patients with symmetric/asymmetric interaural HL. (2) Italics indicate patients with the same HL severity in both ears.

**Table 4:** Audiogram shapes in patients with asymmetric and symmetric interaural HL.

Audiogram shape		The other ear						
		Descending	Flat	Residual	Valley like	Ascending	Total deafness	Other
One ear	Descending	*41 (9)*	0 (21)	0 (8)	0 (6)	0 (1)	0 (2)	0 (13)
	Flat		*16 (5)*	0 (12)	0 (8)	0 (4)	0 (3)	0 (29)
	Residual			*19 (0)*	0 (1)	0 (0)	0 (5)	0 (12)
	Valley like				*1 (0)*	0 (1)	0 (1)	0 (7)
	Ascending					*0 (1)*	0 (0)	0 (2)
	Total deafness						*6 (0)*	0 (4)
	Other							*5 (1)*

(1) The value outside/inside each pair of parentheses represents the number of patients with symmetric/asymmetric interaural HL. (2) Italics indicate patients with the same audiogram shape for both ears.

**Table 5:** Degree of hearing loss in patients (both ears) according to age group.

Age	Degree of hearing loss				
	Mild	Moderate	Moderate to severe	Severe	Profound
Group 1	1 (0.32%)	2 (0.64%)	19 (6.09%)	42 (13.46%)	248 (79.49%)
Group 2	0	12 (20.69%)	12 (20.69%)	9 (15.52%)	25 (43.10%)
Group 3	0	4 (11.76%)	8 (23.53%)	4 (11.76%)	18 (52.94%)
Group 4	0	2 (2.38%)	7 (8.33%)	15 (17.86%)	60 (71.43%)

Group 1 (≤6 years old), group 2 (6.1–12 years old), group 3 (12.1–18 years old), and group 4 (≥18 years old). The value outside each pair of parentheses represents the number of ears with this degree of hearing loss; the value inside each pair of parentheses represents the percentage of ears with this degree of hearing loss relative to the total number of ears in each group.

**Table 6:** Types of audiograms in patients (both ears) with different age groups.

Age	Types of audiograms						
	Descending	Flat	Residual	Valley like	Ascending	Total deafness	Other
Group 1	56 (17.95%)	89 (28.53%)	53 (16.99%)	20 (6.41%)	7 (2.24%)	23 (7.37%)	64 (20.51%)
Group 2	32 (55.17%)	11 (18.97%)	2 (3.45%)	5 (8.62%)	2 (3.45%)	0	6 (10.34%)
Group 3	21 (61.76%)	5 (14.71%)	3 (8.82%)	0	0	1 (2.94%)	4 (11.76%)
Group 4	42 (50.00%)	14 (16.67%)	17 (20.24%)	1 (11.90%)	1 (11.90%)	3 (3.57%)	6 (7.14%)

Group 1 (≤6 years old), group 2 (6.1–12 years old), group 3 (12.1–18 years old), and group 4 (≥18 years old). The value outside each pair of parentheses represents the number of ears with this type of audiogram; the value inside each pair of parentheses represents the percentage of ears with this type of audiogram relative to the total number of ears in each group.

**Table 7:** Interaural hearing symmetry or asymmetry in patients according to age group.

Age	Interaural hearing symmetry or asymmetry			
	Symmetry	Asymmetry		
		Type A	Type B	Type C
Group 1	47 (30.13%)	7 (4.49%)	79 (50.64%)	23 (14.74%)
Group 2	12 (41.38%)	1 (3.45%)	10 (34.48%)	6 (20.69%)
Group 3	9 (52.94%)	0	6 (35.29%)	2 (11.76%)
Group 4	20 (47.62%)	6 (14.29%)	11 (26.19%)	5 (11.90%)

Group 1 (≤6 years old), group 2 (6.1–12 years old), group 3 (12.1–18 years old), and group 4 (≥18 years old). The value outside each pair of parentheses represents the number of patients with interaural hearing symmetry or asymmetry; the value inside each pair of parentheses represents the percentage of patients with interaural hearing symmetry or asymmetry relative to the total number of patients in each group.

**Table 8:** HL severity and audiogram shapes in three pairs of siblings.

		HL severity		Audiogram shape	
		Left ear	Right ear	Left ear	Right ear
Set 1	Older sister	Moderate	Severe	Descending	Other
	Younger sister	Moderate	Moderate	Flat	Flat
Set 2	Older brother	Profound	Profound	Descending	Descending
	Younger brother	Profound	Profound	Descending	Flat
Set 3	Older sister	Moderate to severe	Moderate to severe	Flat	Descending
	Younger brother	Moderate to severe	Moderate to severe	Descending	Flat

## Data Availability

The data used to support the findings of this study are available from the corresponding authors upon request.
